# Natural quorum sensing inhibitors effectively downregulate gene expression of *Pseudomonas aeruginosa* virulence factors

**DOI:** 10.1007/s00253-019-09618-0

**Published:** 2019-03-09

**Authors:** Syed A. K. S. Ahmed, Michelle Rudden, Thomas J. Smyth, James S. G. Dooley, Roger Marchant, Ibrahim M. Banat

**Affiliations:** 1 School of Biomedical Sciences, Ulster University, Coleraine, BT52 1SA UK; 2 Department of Biology, University of York, Wentworth, York, YO10 5DD UK; 3 School of Science, Institute of Technology Sligo, Sligo, Ireland

**Keywords:** Trans-cinnamaldehyde, Salicylic acid, Quorum sensing, Quorum sensing inhibitor, *Pseudomonas aeruginosa*

## Abstract

At present, anti-virulence drugs are being considered as potential therapeutic alternatives and/or adjuvants to currently failing antibiotics. These drugs do not kill bacteria but inhibit virulence factors essential for establishing infection and pathogenesis through targeting non-essential metabolic pathways reducing the selective pressure to develop resistance. We investigated the effect of naturally isolated plant compounds on the repression of the quorum sensing (QS) system which is linked to virulence/pathogenicity in *Pseudomonas aeruginosa*. Our results show that *trans*-cinnamaldehyde (CA) and salicylic acid (SA) significantly inhibit expression of QS regulatory and virulence genes in *P. aeruginosa* PAO1 at sub-inhibitory levels without any bactericidal effect. CA effectively downregulated both the *las* and *rhl* QS systems with *lasI* and *lasR* levels inhibited by 13- and 7-fold respectively compared to 3- and 2-fold reductions with SA treatment, during the stationary growth phase. The QS inhibitors (QSI) also reduced the production of extracellular virulence factors with CA reducing protease, elastase and pyocyanin by 65%, 22% and 32%, respectively. The QSIs significantly reduced biofilm formation and concomitantly with repressed rhamnolipid gene expression, only trace amount of extracellular rhamnolipids were detected. The QSIs did not completely inhibit virulence factor expression and production but their administration significantly lowered the virulence phenotypes at both the transcriptional and extracellular levels. This study shows the significant inhibitory effect of natural plant-derived compounds on the repression of QS systems in *P. aeruginosa*.

## Introduction

A review commissioned by the UK government in 2014 (https://amr-review.org/) predicted that there will be more deaths in the world due to antimicrobial resistance (AMR) than cancer by the year 2050. Antibiotic usage creates an evolutionary stress response in the bacterial population that over time leads to the emergence of resistant strains. Extensive use of antibiotics coupled with the diminished pipeline of new antibiotics has seen a rapid evolution of resistance that has culminated in the development of multi-drug-resistant pathogens that are extremely difficult to treat.

*Pseudomonas aeruginosa*, a Gram-negative opportunistic pathogen, is prevalent in immunocompromised patients suffering from cystic fibrosis (CF) and human immunodeficiency virus (HIV). These bacteria are notorious biofilm producers. The biofilm provides a stratified environment with the core being more anoxic with low bacterial growth and metabolic rates. These metabolically inactive biofilm cells are resistant to β-lactam antibiotics (Anwar and Costerton 1990; Werner et al. 2004) ciprofloxacin, tetracycline and tobramycin (Brown et al. 1988). The biofilm layer also acts as a diffusion barrier reducing the rate of antibiotic penetration, preventing sufficient accumulation of antibiotics and allowing time for expression of resistance genes (Jefferson et al. 2005). In CF, *P. aeruginosa* forms biofilms and readily adapts to the lung environment eventually leading to prolonged inflammation and chronic lung infections that are very difficult to treat using conventional antibiotic methods. In addition, the presence of inducible (MexXY) and constitutive (MexAB-OprM) efflux pumps and the poor permeability of the outer membrane also contribute to the reduced susceptibility of *P. aeruginosa* to a broad range of antibiotics (Aghazadeh et al. 2014, López-Causapé et al. 2017). Effective treatment of *P. aeruginosa* is therefore becoming increasingly challenging with the bacterium showing resistance to even the third and the fourth generations of carbapenems and cephalosporins (Luna et al. 2013; Patel et al. 2014). Therefore, it has become critical to find alternative therapies to successfully clear *P. aeruginosa* infections.

*P. aeruginosa* produces a variety of virulence factors, in a coordinated system, that are reported to enable host colonisation and adaptation (Valderrey et al. 2010; Gellatly and Hancock 2013; Sousa and Pereira 2014). These virulence factors include the production of biofilm, pyocyanin, elastase and rhamnolipid and are under the control of a cell density-dependent signalling regulation known as quorum sensing (QS) (Stover et al. 2000; Lee and Zhang 2014; Sousa and Pereira 2014) The canonical QS system in *P. aeruginosa* includes the *las* and the *rhl* systems both consisting of LuxI type synthases (LasI and RhlI) which produce specific acyl homoserine lactone (AHL) molecules, N-(3-oxododecanoyl)-L-homoserine lactone (3-oxo-C_12_-HSL) and N-butanoyl-L-homoserine lactone (C_4_-HSL). At sufficiently high bacterial concentrations, these AHL molecules then bind to the LuxR type receptors (LasR and RhlR) to form transcriptional activation complexes which regulate the transcription of various genes involved with virulence of *P. aeruginosa* (Papenfort and Bassler 2016).

Interfering with this QS system through application of QS inhibitors (QSI) is a novel therapeutic target that has shown to effectively reduce virulence in opportunistic pathogens. The disruption of QS communication can be achieved through the enzymatic degradation of AHL molecules by lactonases, acylases and oxidoreductases or by using small structural molecules that inhibit the QS signal molecule from binding to its cognate regulatory protein (Morohoshi et al. 2009; Kalia 2013; Kisch et al. 2014; Gupta et al. 2015). A synthetic derivative of a furanone, compound C30 (C30F), has been shown to supress bacterial QS in mice lung models through interference with AHL production (Wu et al. 2004) and through attenuation of QS-regulated production of virulence factors (Hentzer et al. 2003). In the recent past, a range of plant compounds have shown to be effective as anti-QS and anti-biofilm agents (Musthafa et al. 2010; Jayelakshmi et al. 2016; Ouyang et al. 2016; Luo et al. 2017). Kim et al. (2015), using an in silico approach, predicted that natural gingerol could bind to the QS regulator LasR protein. They then demonstrated, using standard assays, a decrease in production of several virulence factors and biofilm formation following exposure to gingerol, consistent with interference of the binding of the cognate signal molecule, 3-oxo-C_12_-HSL, to LasR. Moreover, access to crystal structure of LasI (Gould et al. 2004) and LasR (Bottomley et al. 2007) along with the availability of computer-aided programs like structure-based virtual screening (SB-VS) and molecular docking have been useful in identifying more compounds with potential anti-QS abilities.

A SB-VS experiment unlocked six drugs with LasR structural similarity including salicylic acid (SA), nifuroxazide and chlorzoxazone (Yang et al. 2009). These compounds were able to significantly inhibit QS gene expression and phenotypes in *P. aeruginosa*. In another study, molecular docking results showed that a plant compound, trans-cinnamaldehyde (CA), was able to interact with the LasI substrate binding sites by forming hydrophobic and π-π bonds with phenylalanine-27 and 105, tryptophan-33 and a hydrogen bond with arginine-30 in the LasI synthase (Chang et al. 2014). Since QS is related to several virulence mechanisms in *P. aeruginosa*, therefore the ability of compounds like SA and CA to interfere with the QS system can open the possibility of utilising these as effective anti-QS agents for controlling the pathogenic phenotypes of *P. aeruginosa*.

The QS system allows bacteria to adapt to changing environmental conditions at the population level, with the adaptation mediated at the transcriptional level via regulated expression of the QS genes in response to metabolic and environmental stimuli (Wagner et al. 2003; Scott and Hwa 2011). Understanding the transcriptional expression of the QS genes is therefore essential for understanding the physiology of the cell under QS inhibitory conditions. The current information on the ability of CA and SA to reduce QS activity in *P. aeruginosa* is very limited and has been mostly acquired through crude estimations of virulence proteins or by using high throughput microarray analysis for identifying changes to gene expression (Prithiviraj et al. 2005; Yang et al. 2009). Therefore, in this study, we have used a very robust and MIQE (minimum information for publication of quantitative real-time PCR experiments) compliant reverse transcription quantitative PCR (RT-qPCR) assay, a gold standard for low-medium throughput quantitative expression analysis, to study the changes in transcriptomic profiles when *P. aeruginosa* is subjected to CA and SA treatments at sub-inhibitory concentrations. To correlate the effects of the gene expression on the phenotypic profiles following QSI treatment, the QS-regulated virulence factors rhamnolipid, elastase, protease and pyocyanin were estimated.

## Materials and methods

### Bacterial strains and media

The fully sequenced and widely reported laboratory strain *P. aeruginosa* PAO1 (ATCC 15692) was used in the study. Overnight cultures were prepared from − 80 °C frozen culture stocks in a nutrient-rich LB broth at 37 °C under shaking conditions at 180 rpm. This culture was subsequently used to inoculate proteose-peptone-glucose-ammonium-salts (PPGAS) medium (Zhang and Miller 1992). The bacteria were cultivated in PPGAS medium at 1/5th MIC levels of 2.27 mM CA and 3.62 mM SA in either single or combination treatments. A positive control for QS was included using 10 μM C30F (Skindersoe et al. 2008). All experiments were carried out in biological triplicates. The experimental compounds were purchased from Sigma-Aldrich, UK, unless otherwise stated.

### Minimum inhibitory concentration determination

The minimum inhibitory concentration (MIC) of the test inhibitors against *P. aeruginosa* PAO1 was determined using the resazurin microtiter plate assay (Elshikh et al. 2016) which used the redox indicator resazurin that changed colour from blue to pink in the presence of viable cells. The MIC was determined as the concentration at which there was no colour change following 4 h incubation of the overnight cells with 0.015% resazurin.

### RNA isolation and purity assessment

The cell pellets were collected from different growth phase cultures by spinning them at 13,000×*g* for 2–3 min at room temperature and the RNA extracted using JetGene RNA Purification Kit (Thermo Fisher Scientific). The cells were lysed with occasional vortexing in a buffer solution with 1× TE buffer, 15 mg/ml lysozyme and 20 mg/ml proteinase K (Promega). The samples were then transferred to a 2-ml Lysing Matrix A tube (MP Biomedicals) with β-mercaptoethanol containing RLT buffer (provided in the kit) for enhanced lysis. The contents in the lysing matrix tubes were then homogenised using the FastPrep™ FP 200 cell disrupter at speed 5.5 for 30 s. A double DNA-digestion treatment was done to ensure that the RNA was free of any genomic DNA (gDNA) contamination. The RNA isolated was quantified using the Nanodrop spectrophotometer with A_260_/A_280_ ratio of 1.8–2.1 being considered as pure. The integrity of the samples was checked by agarose gel electrophoresis for presence of two sharp distinct bands representing 23S and 16S rRNA. The integrity was further verified by analysing the samples in an Agilent 2100 Bioanalyzer where RNA Integrity Number (RIN) values greater than 8 were observed for all samples. The RIN is based on a numbering system from 1 to 10 with 1 being the most degraded and 10 being the most intact. The RNA samples were aliquoted and stored at − 80 °C.

### Reverse transcription quantitative polymerase chain reaction

First-strand cDNA was synthesised using Superscript™ Reverse Transcriptase II (Invitrogen). Each reaction mix contained DNase-treated RNA (500 ng), 20–250 ng random primers (Promega), 10 mM dNTPs and RNase free water to make to the reaction volume 15.6 μl. The reactions were heated at 65 °C for 5 min before adding 5× strand buffer, 0.1 M DTT and RNase inhibitor (RNAse out™ Invitrogen) in final concentrations of 1×, 10 μM and 40 units, respectively. The reactions were incubated at 25 °C for 2 min before adding Superscript™ II Reverse Transcriptase (200 units final concentration) (Invitrogen). The RT reactions were carried out at a series of temperature starting with 25 °C for 10 min, 42 °C for 50 min and 70 °C for 15 min. The first-strand cDNA synthesis was performed for all the biological triplicates from each time point. A negative reaction without reverse transcriptase was included in every run. All cDNA samples were stored at − 20 °C prior to use.

The cDNA synthesised was then used as a template for real-time PCR amplification using the ROCHE LightCycler LC480 system with a SYBR-Green probe. Since PCR efficiency is highly dependent on primer specificity, therefore a qPCR calibration curve was generated from each primer set using PAO1 gDNA. Only those primers which gave a calibration curve with a slope value between − 3.1 and − 3.6 that translated into amplification efficiencies of 90–110% were used for PCR quantification. The binding specificity of these primers were also validated post-amplification by generating a melt curve for each primer set with the presence of a single sharp peak eliminating the chances of any non-specific binding.

The qPCR 10 μl reaction mix each contained 2× SYBR Green master mix (1×), forward and reverse primers (1 μM), cDNA template and molecular grade water. Negative controls in form of –RT (no reverse transcriptase) and no template control NTC (no DNA template added) were included to rule out any contamination during the preparation process. A positive control in the form of gDNA was also included. The cut-off values for residual gDNA amplification and NTC were set at greater than 35 and 40 cycles, respectively. The cycling parameters were as follows: initial denaturation at 95 °C for 5 min, 40–50 cycles of denaturation at 95 °C for 10 s, annealing at 59 °C for 10 s, extension at 72 °C for 10 s.

### Reference gene validation

A total of six candidate genes (*gyrB, proC, cysG, rpoD, rpoB* and *16S*) were analysed under inhibitory conditions to assess for the most stable and reliable reference genes for this study. The stability of the six candidate genes were validated under inhibitory conditions using three independent software packages geNorm (Vandesompele et al. 2002), NormFinder (Andersen et al. 2004) and BestKeeper (Pfaffl et al. 2004). The geNorm algorithm measures the stability of the genes based on pairwise variation between one candidate gene and the other genes and was calculated using the online available tool RefFinder (Fu et al. 2013). The NormFinder model considers the intra- and inter-group variation to calculate the stability of the genes using a R-based software excel package (MOMA, Aarhus University Hospital, Denmark). The BestKeeper is a free excel-based tool that correlated the coefficient of the candidate gene with a BestKeeper Index to generate the most stable gene. The genes *rpoD* and *proC* were identified as most stable for use as reference genes in this study by the three algorithms.

### Relative gene expression data analysis

System (LC480 software, version 2)-generated analysis was performed on the real-time PCR data. The threshold values (Cq) from each of the qPCR run were extracted from the LC480 system using the second derivative maximum method (Rasmussen 2001). Data analysis was performed by taking the arithmetic mean of the Cq values of the technical replicates and transferring it into log values to generate the relative quantities (RQ). The RQ values of the target genes were then divided by geometric mean of reference gene RQs (*rpoD* and *proC*) to give normalised relative quantity value (NRQ). The NRQ value was then divided by the experimental calibration which in the experiment was relative expression at early log (6-h) and was set to 1. The output was the calibration normalised ratio (CNRQ) which was used in extrapolating information on the expression profile of the target genes.

### Production of virulence factors

An overnight PAO1 culture was used to inoculate PPGAS medium and incubated for 24 h under continuous shaking at 37 °C. The supernatant was collected, and filter sterilised for use in the following assays:

#### Protease

The amount of LasA protease produced by PAO1 following incubation with and without the inhibitors were estimated by adding 0.1 ml culture supernatant to a reaction mixture containing 0.8% azocasein in 500 μl of 50 mM K_2_HPO_4_ (pH 7) and incubating at 25 °C for 3 h. The reaction was terminated by adding 0.5 ml of 1.5 M HCl and then keeping it on ice for 30 min. The precipitated protein was removed by centrifugation (10,000×*g* for 10 min). NaOH (1 N) was added to the supernatant in equal ratios and the concentration of acid soluble azopeptides measured spectrophotometrically at 440 nm.

#### Elastase

The LasB elastase production was measured by adding 1 ml of the culture supernatant to a 2-ml reaction buffer (100 mM Tris-HCl, 1 mM CaCl_2_) containing the substrate elastin-Congo red and incubating for 3 h at 37 °C with shaking at 180 rpm. The reaction was terminated by adding 2 ml of 0.7 M sodium phosphate buffer (pH 6) and placing it on ice for 15 min. The absorbance of the supernatant was measured at 495 nm.

#### Pyocyanin

The pyocyanin concentration was estimated by adding 7.5 ml filtered supernatant to 4.5 ml of chloroform and vortexed until the colour changed to greenish blue. The samples were centrifuged (10,000×*g* for 10 min) and 3 ml of the resulting blue coloured liquid was transferred to a new tube containing 1.5 ml of 0.2 M HCl and shaken until the blue colour turned to pink. The pink layer was transferred to a cuvette and the absorbance measured at 520 nm. The concentration was calculated in μg/ml by multiplying the absorbance by factor 17.072 (Essar et al. 1990).

### Rhamnolipid extraction and purification

The extraction of rhamnolipid was performed following the method of Smyth et al. (2010). The culture supernatant (50 ml) from PAO1 grown in PPGAS medium for 24 h was acidified to pH 2 and extracted with ethyl acetate three times. The organic solvent containing rhamnolipid was dried with anhydrous MgSO_4_ to remove residual water. Rhamnolipid was isolated from the ethyl acetate solvent in the form of yellow gummy residue after removing the organic solvent in a rotary evaporator. The rhamnolipid crude extract was then purified using solid phase extraction by running the samples through Strata SI-1 Silica (55 μM, 70A) Giga tubes (Phenomenex). After conditioning and removing the impurities from the column with chloroform, rhamnolipids were eluted using chloroform and methanol in ratios of 5:0.3, 5:0.5 and 1:1.

### Rhamnolipid separation and analysis by high-performance liquid chromatography mass spectrometry/mass spectrometry

Analysis of the extracted rhamnolipid mixture was performed using a LCQ™ quadrupole ion trap with a negative electrospray ionisation (ESI) interface connected to a Thermo HPLC Spectra system. A reverse phase C18 column with 5 μm particles was used to separate the rhamnolipids. The parameters included desolvation gas at 65 units and source temperature 250 °C, 20 μl injection volume and 0.5 μl/min flow rate. Two mobile phases were used: HPLC grade water (A) and acetonitrile (B). The rhamnolipid congeners were resolved in a linear gradient mobile phase starting with 70%A:30%B to 30%A:70%B over 50 min and then back to 70%A:30%B for 55 min with a final hold of 5 min. Tandem mass spectrometry was carried out using ESI in a negative mode using collision-induced dissociation (CID) at 35% peak within the MS range of 50–800 *m*/*z*.

### Statistical analysis

All statistical analysis was performed using the GraphPad prism v5.

## Results

### Growth phase-dependent expression of QS genes

**Fig. 1:**
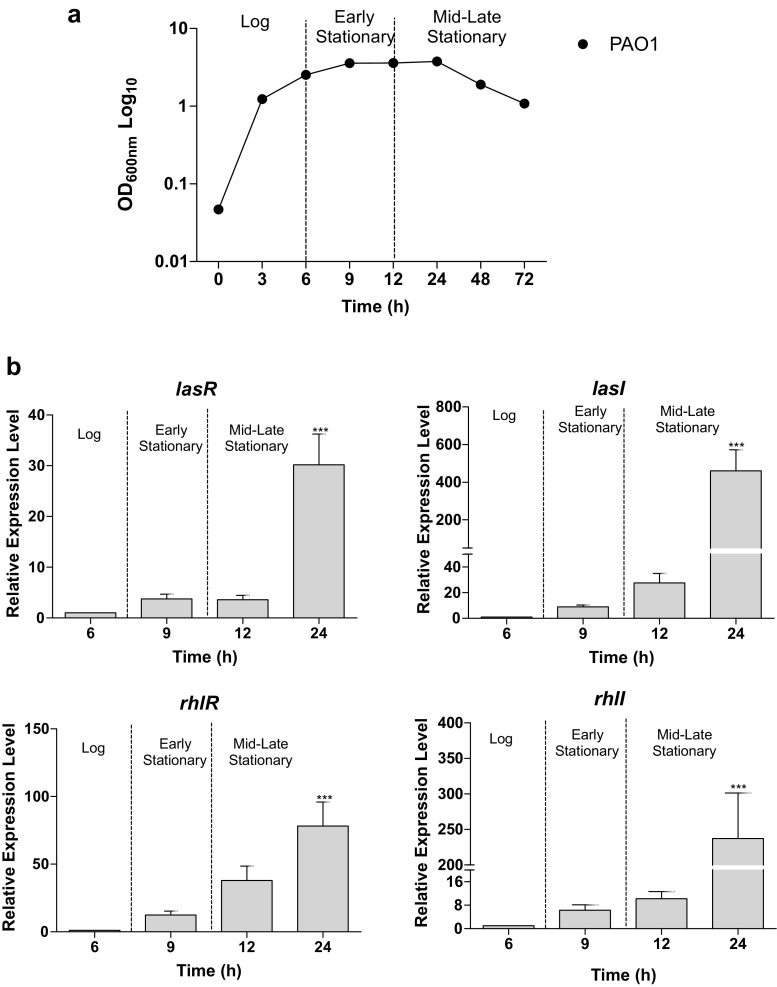
*Las* and *rhl* QS regulatory genes in *P. aeruginosa* PAO1 are differentially expressed in a cell density-dependent manner. **a** Growth of *P. aeruginosa* in phosphate-limited media (PPGAS). **b** Transcriptional expression of QS regulatory genes *lasR, lasI, rhlR* and *rhlI*. Expression levels were quantified by RT-qPCR, relative mRNA levels for target genes were normalised to the geometric mean of two reference genes (*rpoD* and *proC*) and values plotted are the mean calibrator normalised ratios to log phase (6 h). Vertical bars represent S.D. ± (*n* = 3). Data was analysed using one-way ANOVA followed by Dunnett’s multiple comparison test (**p < 0.01, ***p < 0.001)

The effect of the QSIs on the QS system of the fully sequenced laboratory strain *P. aeruginosa* PAO1 (Stover et al. 2000) was investigated by studying the transcriptional expression of the QS synthase and regulatory genes. Both *lasR/lasI* and *rhlR/rhlI* systems were expressed in a cell density-dependent manner with expression levels increasing upon entering the stationary phases of growth (Fig. 1b). Maximum expression levels for all genes were detected in the mid-late stationary phase corresponding with highest cell density. In both *las* and *rhl* systems, the autoinducer synthase genes (*lasI* and *rhlI*) were expressed earlier and at much higher relative concentrations in comparison to their cognate regulatory protein genes (*lasR* and *rhlR*). At high concentrations, LasR and RhlR bind to their cognate N-acyl homoserine autoinducer molecules; the bound complex is then a transcriptional regulator of several genes in *P. aeruginosa*.

**Fig. 2:**
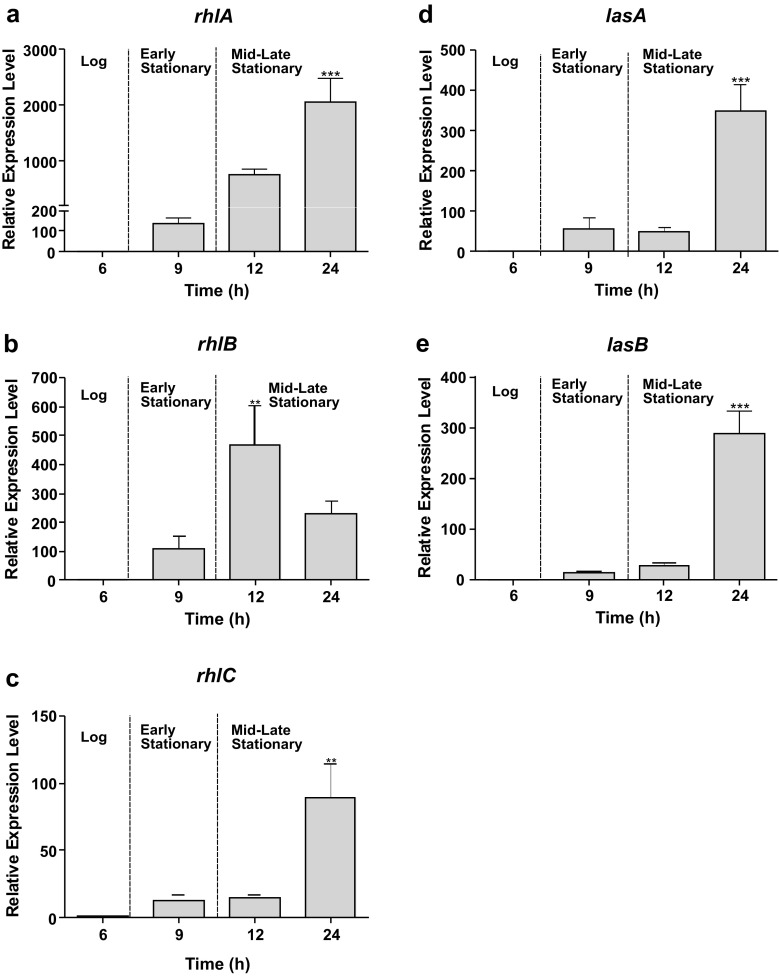
QS-regulated virulence-associated genes are highly expressed in stationary phase in *P. aeruginosa* PAO1. Relative transcript levels of virulence-associated rhamnolipid biosynthetic genes **a***rhlA,***b***rhlB*, **c***rhlC* and exoprotease **d***lasA* and elastase **e***lasB*. Relative mRNA levels for target genes were normalised to the geometric mean of two reference genes (*rpoD* and *proC*) and values plotted are the mean calibrator normalised ratios to log phase (6 h). Vertical bars represent S.D. ± (*n* = 3). Data was analysed using one-way ANOVA followed by Dunnett’s multiple comparison test (*p < 0.05, **p < 0.01, ***p < 0.001)

The QS system regulates productions of most of the *P. aeruginosa* virulence factors including the low molecular weight glycolipids rhamnolipids that are under the direct regulation of the RhlR-RhlI system. The rhamnolipid biosynthetic genes display a differential expression profile where *rhlA* and *rhlB* are expressed earlier relative to *rhlC*, which is only maximally expressed after significant *rhlAB* expression (Fig. 2a–c). The products of *rhlAB* are responsible for the first step in rhamnolipid biosynthesis, which produce mono-rhamnolipids. Mono-rhamnolipids are in turn the substrate for the *rhlC* gene product to produce di-rhamnolipids. The differential sequential expression pattern observed for the rhamnosyltransferases from this data is suggestive of a coordinated regulation based on the substrate availability.

The other virulence-associated genes responsible for the production of the exoprotease LasA, and elastase LasB, were also transcriptionally expressed in a cell density-dependent manner with maximum expression observed in mid-late stationary phase (Fig. 2d–e). The *las*-regulated virulence genes *lasA* and *lasB* were shown to be significantly upregulated during the mid-late stationary phase with expression levels > 300-fold relative to log phase levels (*p* < 0.001).

### Quorum sensing inhibitors effectively downregulate the QS regulatory system

**Fig. 3:**
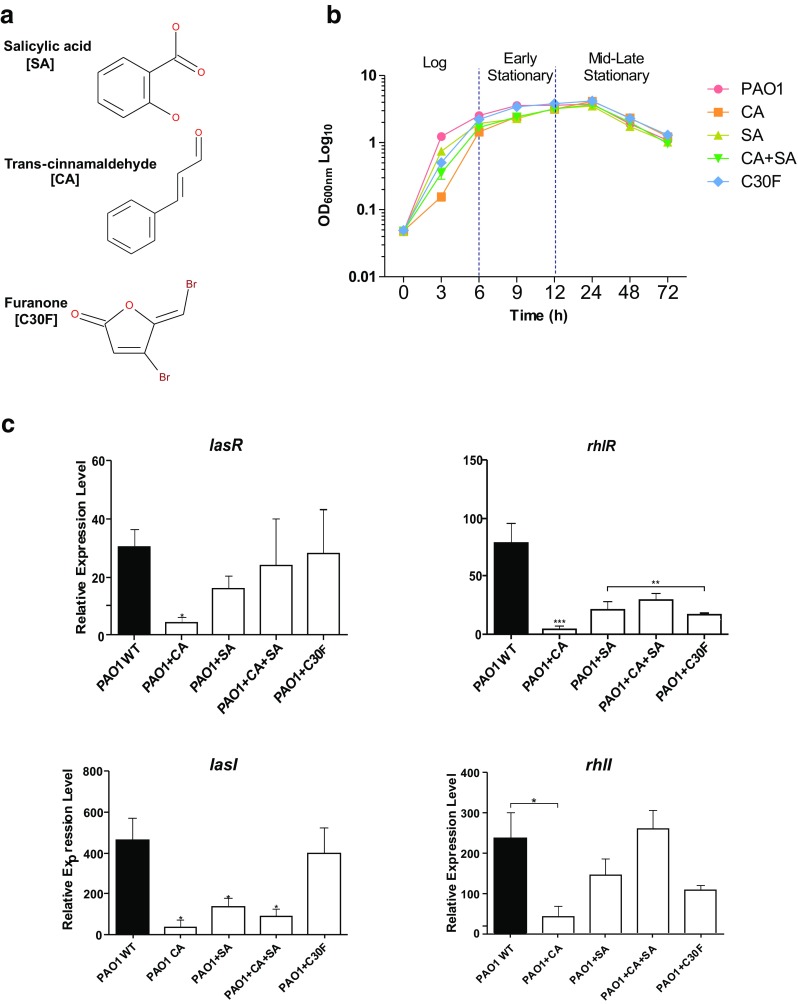
Quorum sensing inhibitors (QSIs) significantly reduce expression of *las* and *rhl* QS systems in *P. aeruginosa*. **a** Molecular structure of the natural QSIs used in this study, salicylic acid (SA), trans-cinnamaldehyde (CA) and positive control furanone C30 (C30F). **b** Growth of *P. aeruginosa* with QSIs at sub-MIC concentrations (SA 3.62 mM, CA 2.27 mM and C30F 10 μM). **c** Relative expression of QS regulatory genes *lasR, lasI*, *rhlR* and *rhlI* with combinations of QSI treatments. Relative mRNA levels for target genes were normalised to the geometric mean of two reference genes (*rpoD* and *proC*). Vertical bars represent S.D. ± (*n* = 3). Data was analysed using two-way ANOVA followed by Bonferroni post-tests (**p < 0.01, ***p < 0.001)

Selectively interfering with QS systems is a novel strategy targeted at disarming virulent opportunistic pathogens such as *P. aeruginosa*. In Gram-negative bacteria, QS is typically mediated by acyl-HSLs and rational analogues have been designed to specifically target these systems. Several phenolic compounds have been be shown to effectively disrupt QS systems in Gram-negative bacteria (Hossain et al. 2017). In this part of the study, we investigated the anti-QS abilities of naturally isolated plant compounds CA, SA and a synthetic furanone compound, C30F, reported to attenuate virulence in *P. aeruginosa* (Fig. 3a) (Hentzer et al. 2003; Yang et al. 2009; Chang et al. 2014). The minimum inhibitory concentration (MIC) of the test compounds CA and SA was determined as 11.35 mM and 18.1 mM, respectively. The use of the quorum sensing inhibitors (QSIs) at the sub-inhibitory concentrations (1/5th MIC) did not affect the growth phenotype of *P. aeruginosa* PAO1 (Fig. 3b). CA treatment resulted in a longer lag phase but reached similar optical densities to untreated PAO1 within 6 h of incubation. Since QS genes were significantly expressed in the stationary phase (Fig. 1b), we tested the effect of the QSIs on the expression of QS-associated regulatory and virulence genes during mid to late stationary phase of growth, when the cell density was at its highest.

CA at sub-inhibitory levels (2.27 mM) significantly (*p* < 0.001) reduced the expression of the QS transcriptional regulatory genes *lasR* and *rhlR* (Fig. 3c)*.* CA caused a 7-fold reduction (*p* < 0.001) in *lasR* gene expression while the difference between the untreated and treated cells was even higher in the LasR-controlled *rhlR* expression with a reduction of 19-fold being observed. CA also effected a significant (*p* < 0.001) reduction in the AHL synthase gene expressions during the stationary phase of growth. The downregulation in the *rhlI* synthase gene following treatment was 6-fold while in *lasI* synthase, it was 13-fold during the late stationary phase.

The second inhibitor tested was the plant hormonal compound SA at sub-inhibitory concentration of 3.62 mM. This also caused inhibition in QS gene expressions but unlike CA, the overall reductions were lower (Fig. 3c). The compound seemed to have a greater inhibitory effect on the *las* QS circuit unlike CA which effectively repressed both *las* and *rhl* QS synthase and regulatory genes. The downregulation in QS transcriptional regulatory genes *lasR* and *rhlR* due to SA treatment was 2-fold and 4-fold, respectively. The transcript levels of the *lasI* synthase gene were three times lower following SA treatment, while there was no significant reduction in expression of the *rhlI* synthase gene in the stationary phase. The behaviour of the *lasR* and *rhlR* regulatory genes determines the expression of virulence-related genes associated with the QS mechanisms in *P. aeruginosa*; therefore, these results suggest that SA would not produce a very high downregulation in QS-regulated virulence gene expressions in comparison to CA.

Although CA and SA when used alone did show reduction in most QS gene transcripts but when used in combination (PAO1 + CA + SA) the results were inconclusive (Fig. 3c). The combination treatment influenced the *lasI* synthase expression where it reduced the transcript level by 5-fold. A similar reduction (3-fold) was also observed in the transcriptional regulator *rhlR* expression. However, the combination treatment did not exert any significant effect on the expression levels of the *lasR* and *rhlI* genes. These results suggest that the inhibitory effects of CA and SA on the QS gene transcriptions were compromised when used in combination.

The positive control C30F produced an expected inhibitory effect on the *rhl* circuit of *P. aeruginosa* during the mid-late stationary phase when used at a concentration of 10 μM (Fig. 3c). The *rhlR* transcript level was reduced 5-fold while the synthase gene *rhlI* was repressed by 2-fold. The compound did not produce any significant inhibition on the transcription levels of the *lasRI* genes.

### Trans-cinnamaldehyde significantly reduces expression of QS-regulated virulence factors

**Fig. 4:**
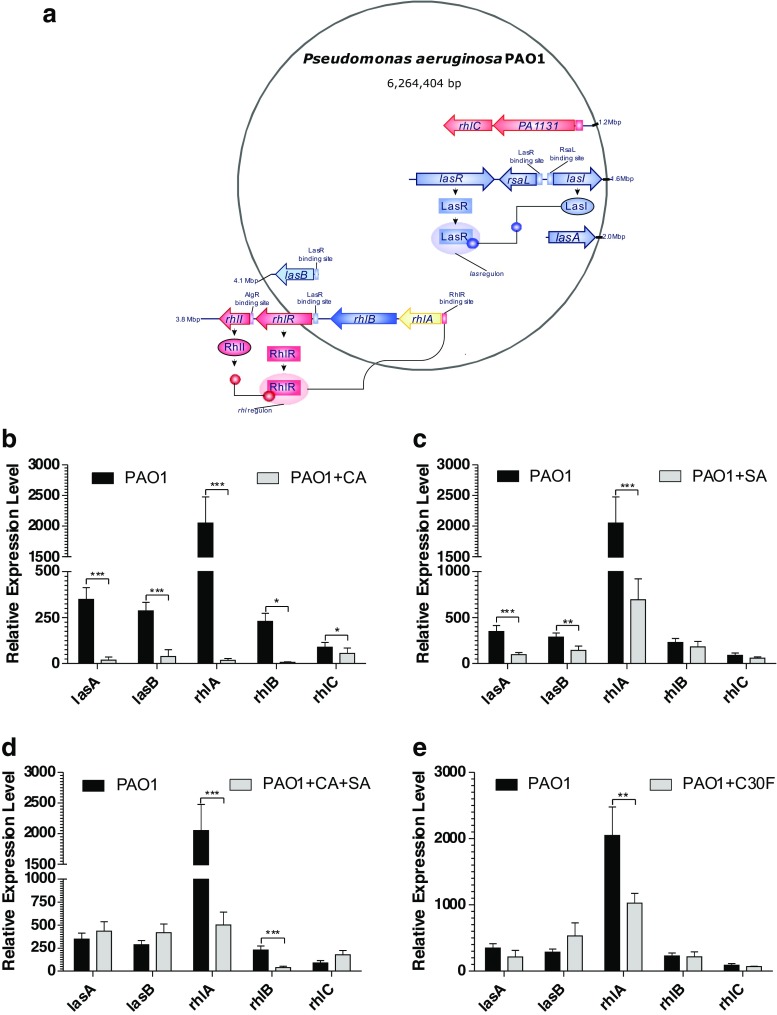
Trans-cinnamaldehyde (CA) significantly reduces gene expression of virulence-associated genes in *P. aeruginosa* PAO1. **a** The schematic representation of the genetic location of las and rhl QS systems in *P. aeruginosa*. Effect of QSIs at the following concentrations of **b** 2.27 mM CA, **c** 3.62 mM SA, **d** 2.27 mM CA + 3.62 mM SA and **e** 10 μM C30F on the transcriptional expression of virulence-associated genes lasA, lasB, rhlA, rhlB and rhlC in *P. aeruginosa* PAO1. Gene expression was quantified at 24 h for both treated and untreated cells, relative mRNA levels for target genes were normalised to the geometric mean of two reference genes (rpoD and proC). Vertical bars represent S.D. ± (*n* = 3). Data was analysed using two-way ANOVA followed by Bonferroni post-tests (*p < 0.05, **p < 0.01, ***p < 0.001)

After investigating the effect of the experimental inhibitors on the QS master genes—*lasRI* and *rhlRI,* the study focused on investigating the inhibitory effects on the *las* and *rhl* QS-regulated set of virulence genes. The target genes selected were *las*-controlled *lasA* protease and *lasB* elastase and *rhl*-regulated genes *rhlA, rhlB* and *rhlC* associated with rhamnolipid production (Fig. 4a). The target gene expression was normalised using validated reference genes, *rpoD* and *proC,* across all culture conditions.

The significant inhibition in *lasRI* expressions in *P. aeruginosa* PAO1 when subjected to CA as seen before affected the mRNA transcript levels of the *lasA* and *lasB* genes (Fig. 4b)*.* The relative expression data showed a 19-fold (*p* < 0.001) reduction in *lasA* gene expression while *lasB* showed a 7-fold (*p* < 0.001) reduction when compared to the untreated cells during the mid-late stationary growth phase. The compound was also effective in highly repressing the expression of the rhamnolipid synthesis *rhlABC* genes during stationary phase (Fig. 4b). The reduction in transcript level of *rhlA* was observed as greater than 100-fold (*p* < 0.001). A significantly high downregulation was also observed in *rhlB* (*p* < 0.05) expression while a 2-fold reduction (*p* < 0.05) was observed in *rhlC* expression.

The ability of SA to repress the las QS genes *lasI* and *lasR* (Fig. 3c) consequently influenced the virulence gene expressions of *lasA* and *lasB*. The transcript levels of *lasA* and *lasB* were reduced by 4-fold (*p* < 0.001) and 2-fold (*p* < 0.01) respectively when treated with 3.62 mM SA (Fig. 4c). It can be hypothesised that the inability of SA to produce an inhibitory effect on *rhlI* synthase gene expression (Fig. 3c) meant there were enough signal molecules to drive the expression of the *rhlABC* genes. Although a 3-fold (*p* < 0.001) reduction was observed in *rhlA* gene expression, partly due to the reduced expression of the *rhlR* regulatory gene, the overall inhibitory effect seemed small as insignificant reductions in *rhlB* and *rhlC* gene expressions were observed with SA at the concentration tested in this experiment (Fig. 4c).

The downregulation in the *lasI* synthase gene when treated with both 2.27 mM CA and 3.62 mM SA did not correlate in a mRNA reduction of the *las*-regulated virulence genes *lasA* and *lasB* (Fig. 4d). However, the ability of the combination treatment to repress the QS regulatory *rhlR* gene caused a significant downregulation (*p* < 0.001) in the *rhlAB* genes. The combination treatment exerted a 4-fold decrease in the *rhlA* gene and 6-fold decrease in the *rhlB* gene expressions compared to the untreated samples. Interestingly, a minor upregulation of 2-fold was observed in the *rhlC* gene.

The inability of C30F to reduce las*-*regulated QS regulatory and synthase gene expressions was only validated in the target gene expression analysis with no reduction being observed in las-controlled virulence *lasA* and *lasB* expressions (Fig. 4e). But C30F’s ability to reduce *rhlRI* had a consequential effect on the expression of *rhlAB* genes with reduction of 2-fold and 3-fold in *rhlA* and *rhlB* gene expressions respectively at late-mid stationary phases.

### QSIs reduce production of extracellular virulence factors at sub-MIC concentrations

#### Biofilm formation

**Fig. 5:**
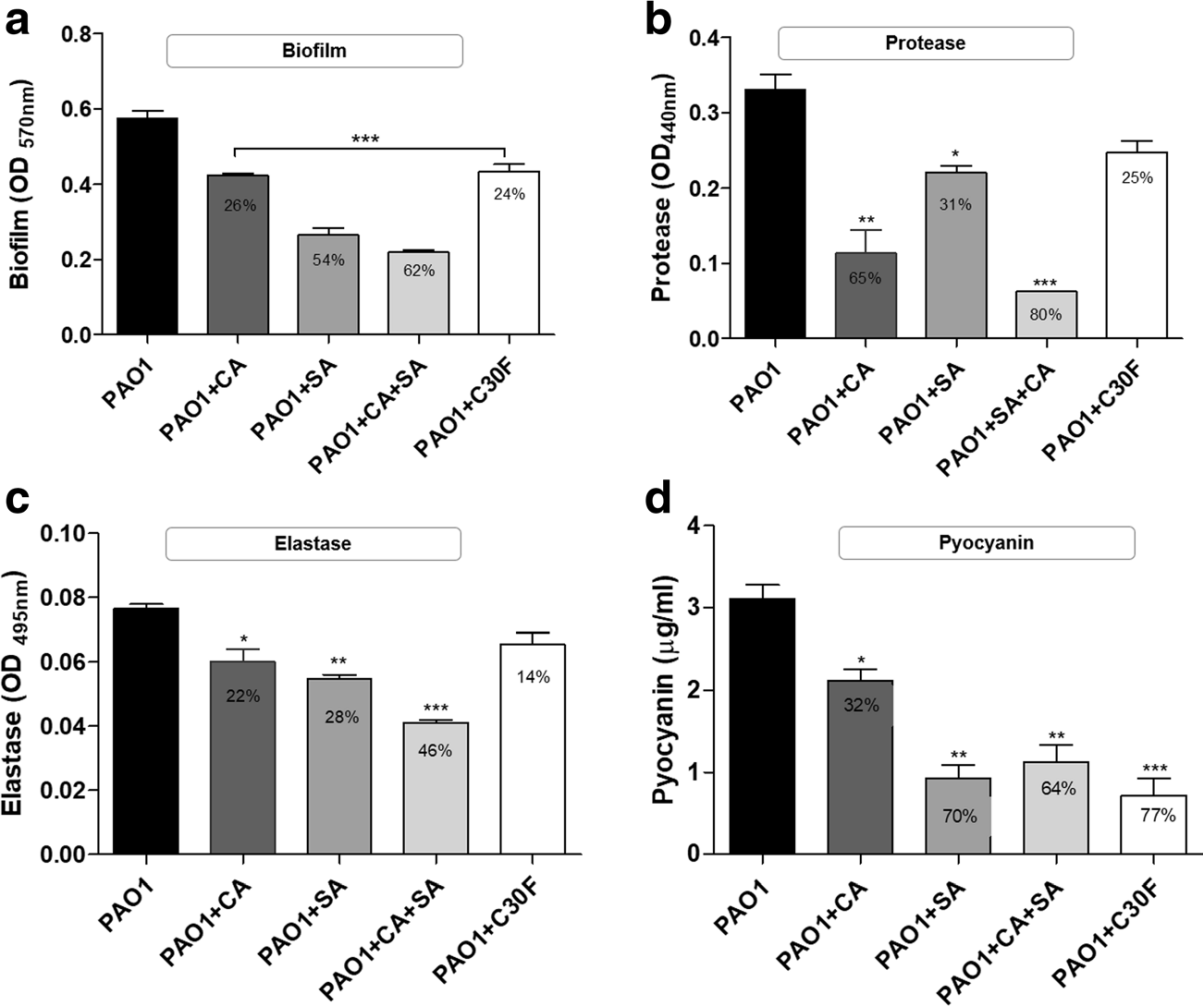
QSIs quantitatively reduce production of extracellular virulence factors in *P. aeruginosa* PAO1. QS-regulated phenotypes **a** biofilm formation and extracellular factors **b** protease, **c** elastase and **d** pyocyanin were significantly disrupted by QSIs. The percentage reductions mentioned were calculated against the untreated PAO1. Error bars represent S.D. ± (*n* = 3). Data was analysed using one-way ANOVA followed by Dunnett’s multiple comparison test (*p < 0.05, **p < 0.01, ***p < 0.001)

The ability of *P. aeruginosa* PAO1 to form biofilm was assessed using a widely employed in vitro model outlined by O’Toole (2011) with slight modifications. The biofilm growth was visualised as a ring of biomass stained with crystal violet at the air-liquid interface. The biofilm formation was evaluated both in the presence and absence of inhibitory compounds, by measuring the absorbance of crystal-violet-stained-adherent-cells solubilised in ethyl acetate at 570 nm from stationary phase cultures (24-h) (Fig. 5a). There were significant (*p* < 0.001) reductions in treated samples compared to the untreated PAO1. In comparison to CA, SA was more effective in reducing the formation of biofilm in the microtiter wells with an absorbance reduction of 54% compared to the untreated cells. CA was also effective, to a lesser degree, with reductions of 26%. The combined use of CA and SA was the most effective of the treatment methods with a reduction of 62%. The positive control C30F in comparison was the least effective (24%) in reducing biofilm formation.

#### LasA protease

There were significant reductions in LasA protease activity in the QSI-treated cells as estimated from absorbance reading resulting from azo dyes released into the medium due to proteolytic cleavage of the substrate azocasein. In the presence of SA, the OD_440_ dropped from 0.3 to 0.1 to account for a 31% reduction (*p* < 0.05) in absorbance reading while CA treatment gave an even higher reduction of 65% (*p* < 0.01). The combination QSIs (CA + SA) treatment produced the highest reduction in protease production with a reduction of 80% (*p* < 0.001) absorbance being observed when compared to the untreated PAO1 (Fig. 5b).

#### LasB elastase

The elastase production was estimated through absorbance measurement of Congo red following cleavage of elastin-Congo red substrate by the enzyme elastase produced by *P. aeruginosa*. In presence of inhibitors CA and SA, the OD_495_ decreased from 0.08 to 0.06 and 0.05 respectively giving subsequent reduction percentages of 22% (*p* < 0.01) and 28% (*p* < 0.05) (Fig. 5c). The combination treatment was again the most effective in reducing absorbance with reduction of 46%. However, like the protease assay, C30F was the least effective, confirming earlier results of its reduced inhibitory effects on *lasA* and *lasB* gene expressions.

#### Pyocyanin

Pyocyanin production is regulated by the *rhl* QS via PQS; hence, the measurement of pyocyanin inhibition is also a good indicator of the effectiveness of the tested compounds as QS inhibitors in *P. aeruginosa*. The pyocyanin concentration decreased from 3.1 μg/ml to 2.1 μg/ml and 0.922 μg/ml in the presence of CA and SA, respectively. When used together, the inhibitors decreased the yield by 64% (1.1 μg/ml) (Fig. 5d).

#### Rhamnolipid estimation

**Fig. 6:**
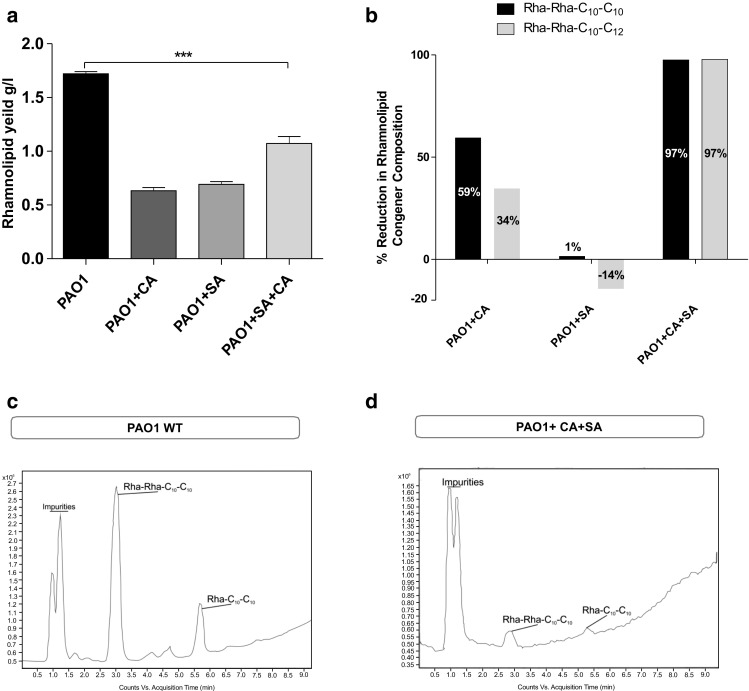
QSIs significantly reduce *rhl*-regulated rhamnolipid production *P. aeruginosa*. **a** The QSIs reduces the rhamnolipid crude yield significantly compared to the untreated PAO1 cells. **b** % reduction in the two main RL congeners Rha-C_10_-C_10_ and Rha-Rha-C_10_-C_10_ relative to untreated *P. aeruginosa* PAO1. RL congeners were studied by HPLC-MS/MS. The HPLC chromatograms of **c** untreated *P. aeruginosa* PAO1 and **d***P. aeruginosa* PAO1 treated with trans-cinnamaldehyde (CA) and salicylic acid (SA)

The administration of the QS inhibitors caused a reduction in the yield of rhamnolipid with CA and SA both producing a drop in crude weight from 1.72 g/l to 0.7 g/l (approximately) (Fig. 6a). Rhamnolipids are produced as congeners containing one to two rhamnose sugar moieties giving the compounds their distinctive properties (Chen et al. 2010; Chen et al. 2013). The structural composition of rhamnolipid produced in the presence of the QSIs was studied by analysing the purified crude sample using high-performance liquid chromatography mass spectrometry/mass spectrometry (HPLC-MS/MS) method. The inhibitor treatment when used alone did not affect the composition of the rhamnolipid with the congener profile resembling the untreated PAO1 sample (Fig. 6c). But in the combination treatment (PAO1 + CA + SA), only two rhamnolipid congeners were detected by the HPLC-MS/MS method (Fig. 6d) compared to six in the untreated sample. This combination treatment also effected the maximum reduction in the relative amount of rhamnolipid obtained from a 50-ml culture supernatant. The two predominant congeners identified in all the samples were Rha-Rha-C_10_-C_10_ (*m*/*z* 649) and Rha-Rha-C_10_-C_12_ (*m*/*z* 677). The mono-rhamnolipid detected in greatest abundance was Rha-C_10_-C_10_. When the MS data for the two common di-rhamnolipid congeners were compared to untreated sample, the combination treatment and CA treatment showed marked differences (Fig. 6b). SA treatment did not show any noticeable difference in relative quantification of the congeners although it resulted in a decrease in crude weight.

## Discussion

### Trans-cinnamaldehyde is more effective than salicylic acid in reducing expression of QS genes

This study is consistent with previous research showing that natural QSIs significantly modulate transcriptional expression of QS regulatory and virulence-associated genes during stationary phase in *P. aeruginosa* PAO1. CA effectively inhibited the expression of both *las* and *rhl* QS systems. Both the regulatory proteins (LasR and RhlR) and the AHL synthases (LasI and RhlI) were significantly repressed with CA. Downregulation of both these QS systems correlated with repression of their virulence-associated genes. The exact mechanism of action is unknown for these compounds. Several natural and synthetic antagonists have been described for LasR; however, the relative instability of LasR::antagonist complexes has limited biochemical characterisation in vitro. Recently, O’Reilly et al. (2018) used potent agonists rather than known antagonists to stabilise LasR in vitro. They were able to develop a focused library of agonists based on previous tri-phenyl ligands, resulting in several new LasR::agonist complexes available in/from the PDB. From these structures, O’Reilly et al. (2018) determined an important functional role for a flexible loop in the ligand binding domain (LBD), previously unknown, which upon ligand binding promotes specific conformational changes that seals the ligand binding pocket from solvent and directs the DNA binding domain (DBD) to form a transcriptional activation complex. This suggests a plausible mechanism by which agonists stabilise and antagonists destabilise LasR. These structures provide essential information for the fundamental understanding of how LuxR type receptors bind to their cognate autoinducers.

We hypothesise that CA and SA act as QS antagonists. Previously, molecular docking studies have suggested CA to interact with the LasI protein (Chang et al. 2014). LasI synthase produces 3-oxo-C_12_-HSL which is the ligand for LasR. RhlI is 47% homologous with LasI (Chang et al. 2014; Gökalsın et al. 2017) and a similar mechanism of action is interpreted in AHL production. In general, LuxI synthases catalyses the transfer of an acyl group bound to acyl carrier protein (ACP) from fatty acid bio-synthesis to S-adenosyl–L-methionine (SAM) (Churchill and Chen 2011) which then undergoes lactonization to form the N-acyl–homoserine lactone. CA is predicated to bind in the SAM binding pocket of LasI, thus preventing SAM binding and subsequent 3-oxo-C_12_-HSL synthesis. In the absence of AHL, LasR will not dimerize and therefore cannot bind to DNA. These interactions could modulate the QS autoinducer levels. The LasI::3-oxo-C_12_-HSL complex regulates the expression of many downstream genes including *lasI* and *rhlI*. Since we showed CA reduces *lasI* expression and previously CA has shown to reduce signal molecule concentration (Chang et al. 2014), we suggest that the intracellular concentration of autoinducer signal molecules was not sufficient to trigger the activation of genes involved with rhamnolipid and protease synthesis as shown in this study.

To date, there is no crystal structure for RhlR, its inherent instability in vitro has proven intractable to crystallisation and biochemical characterisation. Based on similarity, mechanistic interpretations of LasR with AHL ligands and inhibitors are expected to extend to RhlR. However, RhlR remains a viable QS target for developing targeted inhibitors, *lasR* mutants are frequently isolated from cystic fibrosis patients suggesting the redundancy of LasR as a master regulatory in chronic CF infections (Feltner et al. 2016).

The reductions in *lasRI* and *rhlRI* expressions from CA treatments were correlated by assessing the activity of *las*-regulated elastase and protease and *rhl*-regulated pyocyanin and rhamnolipid productions. CA at sub-inhibitory concentrations caused a significant decrease in elastase (22%) and protease (65%) activities. Pyocyanin, which is a good indicator of *rhlI* inhibition (Chang et al. 2014), showed a decrease of 32% with CA. These reductions were comparable to other findings with cinnamaldehyde as QSI in the literature (Brackman et al. 2008; Brackman et al. 2011). Although CA was not able to abolish rhamnolipid production, the treatment caused a decrease in rhamnolipid yield with the two main di-rhamnolipid congeners Rha-Rha-C_10−_C_10_ and Rha-Rha-C_10_-C_12_ levels reduced by 59% and 34% respectively compared to that of untreated cells. The inhibition at post-translational levels of these virulence factors complemented the reverse transcription quantitative polymerase chain reaction (RT-qPCR) data from this study where we observed significant reductions in *lasA*, *lasB*, *rhlA*, *rhlB* and *rhlC* expressions following CA treatment.

SA unlike CA did not produce the same level of inhibition on the transcriptional profiles of the *lasRI* and *rhlRII* genes with 2–4-fold reduction in mRNA levels being observed in the treated samples compared to untreated controls. The binding affinity of SA to the LasR protein (Yang et al. 2009) possibly promoted conformational changes in the LasR-(3-oxo-C_12_-HSL) complex thereby causing a reduced expression of downstream genes. Due to the QS hierarchical arrangement, *rhlR* expression can be regulated by *lasR*; hence, the highest inhibition in QS regulatory expression with SA was seen in *rhlR*. This was in agreement with a previously reported study where SA was shown to reduce *rhlR* expression in *P. aeruginosa* (Yang et al. 2009). The decreased expression in *las* QS genes consequently repressed *lasA* and *lasB* levels supporting the findings of Prithiviraj et al. (2005) using SA. Since SA did not lead to an inhibitory effect on the overall *rhl* regulon, significant downregulation was not observed in the *rhlB* and *rhlC* genes. El-Mowafy et al. (2014) reported SA rich aspirin could cause significant downregulation in the *lasRI* and *rhlRI* expressions. The findings however do not fully agree with the results from this study. The study with aspirin (El-Mowafy et al. 2014) used only one reference gene, *rpoD,* for data normalisation along with a higher concentration of the inhibitor thereby giving slightly different results. Although SA did not show a profound effect at the transcriptional level, it seemed to be effective at the translational level. This can be hypothesised from this study considering higher reductions in virulence proteins elastase and protease were observed in the semi-quantitative assays following SA treatment. Reduction of these proteases when *P. aeruginosa* were supplemented with SA had been previously reported in a couple of studies with inhibition ranging between 40 and 80% (Prithiviraj et al. 2005; El-Mowafy et al. 2014). The choice of semi-quantitative assay and the selection of growth medium were perhaps responsible for the large inhibitory range being observed within the results published in the literature (Duan and Surette 2007). The choice of media is very important as the production of secondary metabolites can be influenced by growth limiting factors present in the medium. However, SA had a negligible effect on the *rhl-*controlled rhamnolipid production with HPLC results being similar to the untreated sample. This complemented the qPCR findings where minimal reduction was seen in the rhamnolipid biosynthesis gene expressions. Moreover, the unavailability of the RhlR crystal structure makes it difficult to predict the possible interaction sites for these inhibitors.

### The combination treatment of CA and SA does not show significant inhibitory effect on QS gene expressions

Having ascertained the potential of CA and SA to repress QS-regulated gene expressions and virulence factor production when used separately, the effect of combination treatment was investigated. Even though CA and SA have different QS targets, in the form of LasI and LasR respectively, expression profiles suggested that the combination treatment was not very effective at the transcriptional level. Noticeable downregulation was observed in *rhlR* which subsequently affected the expression of the rhamnolipid genes, further supporting the view that inhibitors targeting transcriptional regulators can be a potential drug target for reducing bacterial virulence. At post-translational level, the combination treatment was successful in reducing the *rhl-*regulated production of pyocyanin and rhamnolipid. The HPLC-MS/MS analysis showed negligible presence of rhamnolipids strengthening the idea that the effect of the combination treatment was strongly at the translational level. A computational model study of LuxI/LuxR QS suggested that LuxR competitive inhibitor, unlike LuxR non-competitive inhibitor, can display antagonistic effects when used in combination with a LuxI inhibitor (Anand et al. 2013). Therefore, if an analogy is drawn with SA targeting LasR through competitive inhibition, then some of the inhibitory potential of LuxI-type inhibitor CA can be attenuated. However, the mechanism by which this could happen is not known and was beyond the scope of current work. A better understanding on how the inhibitors bind to the target proteins will help to elucidate the lower inhibitory effects observed at expression levels with the combination treatment especially when we consider that significant downregulation was observed with CA alone.

With antibiotics fast losing their efficacy, alternative strategies are imperative. The sole use of QS inhibitors is unlikely to completely eradicate the bacterial infection and there would be legitimate concerns around potential toxicity of high concentrations of cinnamaldehyde where maximum permissible levels in foodstuffs have already been determined (Shreaz et al. 2016). However, since the inhibitors reduce the virulence phenotypes and weaken the bacterial biofilms, this would allow the host innate immunity and externally administered antimicrobial compounds to function more effectively. Synergistic enhancement of antibiotics by administration of sub-inhibitory quorum quenching compounds is a potentially exciting future development but little is known about such effects at the molecular level. Our system provides a suitable model system for future studies aimed at elucidating these mechanisms and should contribute to extending the useable life span of current drugs.
